# Local Determination of Comb Mode Number in Remote Optical Clock Comparison

**DOI:** 10.3390/s26175360

**Published:** 2026-08-25

**Authors:** Hao Liu, Haochen Tian, Bingkun Lu, Qiang Wang, Yige Lin

**Affiliations:** 1 Institute of Precision Optical Engineering, School of Physics Science and Engineering, Tongji University, Shanghai 200092, China; 2 Division of Time and Frequency Metrology, National Institute of Metrology, Beijing 100029, China; 3 Hefei National Laboratory, Hefei 230088, China; 4 Key Laboratory of Time Frequency and Gravity Primary Standard, State Administration for Market Regulation, Beijing 100029, China; 5 Beijing Key Laboratory of Quantum Metrology Technology and Instruments, Beijing 100029, China

**Keywords:** optical clock comparison, absolute frequency measurement, optical frequency comb

## Abstract

Optical clocks and their associated frequency comparison networks are exceptionally sensitive to microscopic physical variations and thus capable of measuring fundamental physical quantities across diverse applications. In frequency comparison networks, the optical frequency combs (OFCs) act as accurate frequency sensors designed to translate terahertz optical signals down to radio frequency signals for accurate frequency counting. The number of OFC’s comb modes that beat with clock and transfer lasers during remote frequency comparison is indispensable for the calculation of their frequency difference. In this work, with the assistance of a 1542 nm cavity-stabilized laser at Changping campus, the number of comb modes is locally determined with sufficient uncertainty by stepping the comb’s repetition rate. This approach bypasses the technical challenges of simultaneous operation of optical clocks, OFC and fiber-link noise cancelation during long-distance comparison, providing a reliable prerequisite for remote optical clock comparisons.

## 1. Introduction

Optical clocks exhibit substantial promise as the next-generation frequency standards owing to their outstanding frequency uncertainty performance. Frequency ratio measurements between optical clocks—also referred as optical clock comparison—and their associated networks function as ultra-sensitive quantum sensors capable of resolving minute physical perturbations enable measurement of variations in fundamental physical constants [1,2,3], detecting gravitational waves [4,5], searching for dark matter [6,7,8,9,10], and test of relativity with high precision [11,12].

Comparisons of optical clocks with different transitions, or remote comparisons with the same transition, require OFCs whose development has revolutionized optical metrology [13,14,15,16,17,18]. In this scenario, OFCs act as precise optical sensors to translate terahertz optical signals to radio frequencies, thereby enabling the measurement of optical frequencies and their ratios. In 2015, Takamoto et al. utilized an Er:fiber OFC to measure the frequency ratio among three local optical clocks with distinct transitions and one remote Sr optical clock [14]. Lisdat et al. compared two Sr optical clocks separately located at Physikalisch-Technische Bundesanstalt (PTB) and Systèmes de Référence Temps-Espace (SYRTE). Connected by a 1415 km fiber link, this implementation of two OFCs verified the agreement of two clocks at an uncertainty of 5 × 10^−17^ [16]. In 2025, JILA and NIST utilized an Er:fiber optical frequency comb and an Er/Yb:glass comb [19] to transfer the stability of a cryogenic silicon cavity [20] to the 1157 nm clock laser and the 1070 nm clock laser at NIST. A Ti:sapphire comb was utilized to measure the frequency ratio among the ^27^Al^+^ ion clock, ^171^Yb optical lattice clock, and ^87^Sr optical lattice clock. The total fractional uncertainties were all at or below 3.2 × 10^−18^ [21]. Recently, our group demonstrated a remote frequency comparison between two ^87^Sr optical lattice clocks located at the Changping and Hepingli campuses via a 58 km noise-canceled fiber link [22,23]. Two OFCs and a 1542 nm transfer laser were employed to remotely link the two optical clocks. In this work, calculation of the frequency difference between the two optical clocks required the determination of the numbers of comb modes of OFCs that beat with the 1542 nm transfer laser. The feasible way of obtaining these key numbers relies on a wavemeter to coarsely measure the frequency of the 1542 nm transfer laser to identify the candidate numbers of the OFC’s comb modes that beat with the laser at Changping campus. Simultaneously keeping two atomic systems in optical clocks, the noise-cancelled fiber link and two OFCs running, these candidates are then cross-referenced against the OFC at Hepingli campus to identify the unique integral number of the OFC’s comb mode. The overall complexity would be greatly reduced if these numbers could be determined at a single location—either at the Changping campus or the Hepingli campus—without running the atomic systems.

In this work, we present an approach to locally determine the numbers of OFC’s modes that beat with the clock laser and the transfer laser during long-distance optical clock comparisons without the requirement of the simultaneous operation of two optical clocks and two OFCs. To this end, we calculate the numbers of comb modes and the absolute frequency for both the 698 nm clock laser and a 1542 nm cavity-stabilized laser by changing the repetition rate of the OFC. Then the absolute frequency of the transfer laser can be obtained by beating it with the 1542 nm cavity-stabilized laser. Finally, the number of comb modes of both OFCs at Changping and Hepingli that beat with the transfer laser can be determined. This method reduces the technical challenges associated with the simultaneous operation of two optical clocks, OFCs and noise-canceled fiber link during long-distance comparison, thereby providing a reliable prerequisite for remote optical clock comparisons.

## 2. Principle

In this work, two methods to determine the absolute frequency of the laser under test (LUT), which subsequently yields the number of OFC’s comb modes that beat with the LUT, are utilized [24]. The first approach is to reference the OFC to the LUT and then measure its repetition rate. In this scenario, both carrier-envelope offset frequency (*f*_ceo_) and comb-LUT beat (denoted as *f*_b_, referring to the beat note between LUT and the *n*-th comb mode of the OFC) are phase-locked to RF (radio frequency) references. The repetition rate of the OFC is recorded within a certain measurement time, yielding *f*_rep1_. According to the comb equation, the frequency of the LUT, *ν*_LUT_, is expressed as follows:

(1) $$\nu_{\mathrm{LUT}}=N\cdot f_{\mathrm{rep}1}+f_{\mathrm{ceo}}+f_{b1}$$

where *N* is the comb mode number of the *n*-th comb mode. In Equation (1), *f*_ceo_ and *f*_b1_ are governed by the RF reference. Then the (*n* − 1)-th, (*n* − 2)-th, (*n* − 3)-th and (*n* − 4)-th comb mode of the OFC is subsequently locked to the LUT, yielding *f*_rep2_, *f*_rep3_, *f*_rep4_ and *f*_rep5_. It is practical to keep *f*_ceo_ and *f*_b_ fixed during the five measurements.

(2) $$\nu_{\mathrm{LUT}}=(N-1)\cdot f_{\mathrm{rep}2}+f_{\mathrm{ceo}}+f_{b2}$$

(3) $$\nu_{\mathrm{LUT}}=(N-2)\cdot f_{\mathrm{rep}3}+f_{\mathrm{ceo}}+f_{b3}$$

(4) $$\nu_{\mathrm{LUT}}=(N-3)\cdot f_{\mathrm{rep}4}+f_{\mathrm{ceo}}+f_{b4}$$

(5) $$\nu_{\mathrm{LUT}}=(N-4)\cdot f_{\mathrm{rep}5}+f_{\mathrm{ceo}}+f_{b5}$$

Through Equations (1)–(5), we obtain the comb mode numbers as follows:

(6) $$N=\frac{f_{\mathrm{rep}2}}{f_{\mathrm{rep}2}-f_{\mathrm{rep}1}}$$

(7) $$N-1=\frac{f_{\mathrm{rep}3}}{f_{\mathrm{rep}3}-f_{\mathrm{rep}2}}$$

(8) $$N-2=\frac{f_{\mathrm{rep}4}}{f_{\mathrm{rep}4}-f_{\mathrm{rep}3}}$$

(9) $$N-3=\frac{f_{\mathrm{rep}5}}{f_{\mathrm{rep}5}-f_{\mathrm{rep}4}}$$

Multiple results from Equations (6)–(9) allow us to validate the correctness of the measured comb mode number. Assuming *u*(*f*_rep1_) ≈ *u*(*f*_rep2_), the uncertainty of the measured comb mode number *N* is governed by the following derivation:

(10) $$\begin{array}{ll} u(N_{n}) & =\sqrt{{\left[\left|\frac{\partial N_{n}}{\partial f_{\mathrm{rep}1}}\right|\cdot u(f_{\mathrm{rep}1})\right]}^{2}+{\left[\left|\frac{\partial N_{n}}{\partial f_{\mathrm{rep}2}}\right|\cdot u(f_{\mathrm{rep}2})\right]}^{2}}\\ & =\frac{\sqrt{{\left[f_{\mathrm{rep}2}\cdot u(f_{\mathrm{rep}1})\right]}^{2}+{\left[f_{\mathrm{rep}1}\cdot u(f_{\mathrm{rep}2})\right]}^{2}}}{{\left(f_{\mathrm{rep}1}-f_{\mathrm{rep}2}\right)}^{2}}\\ & \approx \frac{\sqrt{2}\cdot f_{\mathrm{rep}1}\cdot u(f_{\mathrm{rep}1})}{{\left(f_{\mathrm{rep}1}-f_{\mathrm{rep}2}\right)}^{2}} \end{array}$$

If it is required to satisfy *u*(*N*) < 1, given that *f*_rep1_ = 200 MHz and the difference between *f*_rep1_ and *f*_rep2_ is around 100 Hz, then *u*(*f*_rep1_) < 0.035 mHz must be achieved. This indicates that the statistical uncertainty of the measured *f*_rep_ needs to be reduced to <0.035 mHz through averaging during the measurements.

The second approach is to reference the OFC to the RF reference and record the LUT-comb beat. In this scenario, the *f*_ceo_ and the repetition rate of the OFC are phase-locked to RF references. The comb-LUT beat, *f*_b1_, is recorded within a certain measurement time. Similarly, the repetition rate of the comb is locked to the other four different frequencies (*f*_rep2_, *f*_rep3_, *f*_rep4_ and *f*_rep5_) so that LUT beats with the (*n* + 1)-th, (*n* + 2)-th, (*n* + 3)-th and (*n* + 4)-th comb modes of the OFC generating *f*_b2_, *f*_b3_, *f*_b4_ and *f*_b5_. In practice, the repetition rates can be selected to ensure that five LUT-comb beats fall within a similar frequency range.

(11) $$\nu_{\mathrm{LUT}}=N\cdot f_{\mathrm{rep}1}+f_{\mathrm{ceo}}+f_{b1}$$

(12) $$\nu_{\mathrm{LUT}}=(N+1)\cdot f_{\mathrm{rep}2}+f_{\mathrm{ceo}}+f_{b2}$$

(13) $$\nu_{\mathrm{LUT}}=(N+2)\cdot f_{\mathrm{rep}3}+f_{\mathrm{ceo}}+f_{b3}$$

(14) $$\nu_{\mathrm{LUT}}=(N+3)\cdot f_{\mathrm{rep}4}+f_{\mathrm{ceo}}+f_{b4}$$

(15) $$\nu_{\mathrm{LUT}}=(N+4)\cdot f_{\mathrm{rep}5}+f_{\mathrm{ceo}}+f_{b5}$$

Equations (11)–(15) show the relationship between the frequency of the LUT and the *f*_b_ signal. Solving Equations (11)–(15), one obtains

(16) $$N=\frac{f_{b1}-f_{b2}-f_{\mathrm{rep}2}}{f_{\mathrm{rep}2}-f_{\mathrm{rep}1}}$$

(17) $$N+1=\frac{f_{b2}-f_{b3}-f_{\mathrm{rep}3}}{f_{\mathrm{rep}3}-f_{\mathrm{rep}2}}$$

(18) $$N+2=\frac{f_{b3}-f_{b4}-f_{\mathrm{rep}4}}{f_{\mathrm{rep}4}-f_{\mathrm{rep}3}}$$

(19) $$N+3=\frac{f_{b4}-f_{b5}-f_{\mathrm{rep}5}}{f_{\mathrm{rep}5}-f_{\mathrm{rep}4}}$$

Similarly, multiple results from Equations (16)–(19) allow us to validate the correctness of the measured comb mode number. Assuming *u*(*f*_b1_) ≈ *u*(*f*_b2_), the uncertainty of the measured comb mode number *N* is as follows:

(20) $$\begin{array}{ll} u(N_{n}^{\prime }) & =\sqrt{{\left[\left|\frac{\partial N_{n}}{\partial f_{b1}^{\prime }}\right|\cdot u(f_{b1}^{\prime })\right]}^{2}+{\left[\left|\frac{\partial N_{n}}{\partial f_{b2}^{\prime }}\right|\cdot u(f_{b2}^{\prime })\right]}^{2}}\\ & =\sqrt{\frac{u^{2}(f_{b1}^{\prime })+u^{2}(f_{b2}^{\prime })}{{\left(f_{\mathrm{rep}1}^{\prime }-f_{\mathrm{rep}2}^{\prime }\right)}^{2}}}\\ & \approx \frac{\sqrt{2}\cdot u(f_{b1})}{f_{\mathrm{rep}1}^{\prime }-f_{\mathrm{rep}2}^{\prime }} \end{array}$$

This indicates that the statistical uncertainty of the measured *f*_b_ needs to be reduced to <71 Hz by averaging over five measurements to ensure *u*(*N*) < 1. Once the comb mode number is determined, the absolute frequency of LUT can be calculated using Equation (1) or (11) in either case.

## 3. Experimental Setup and Results

**Figure 1:**
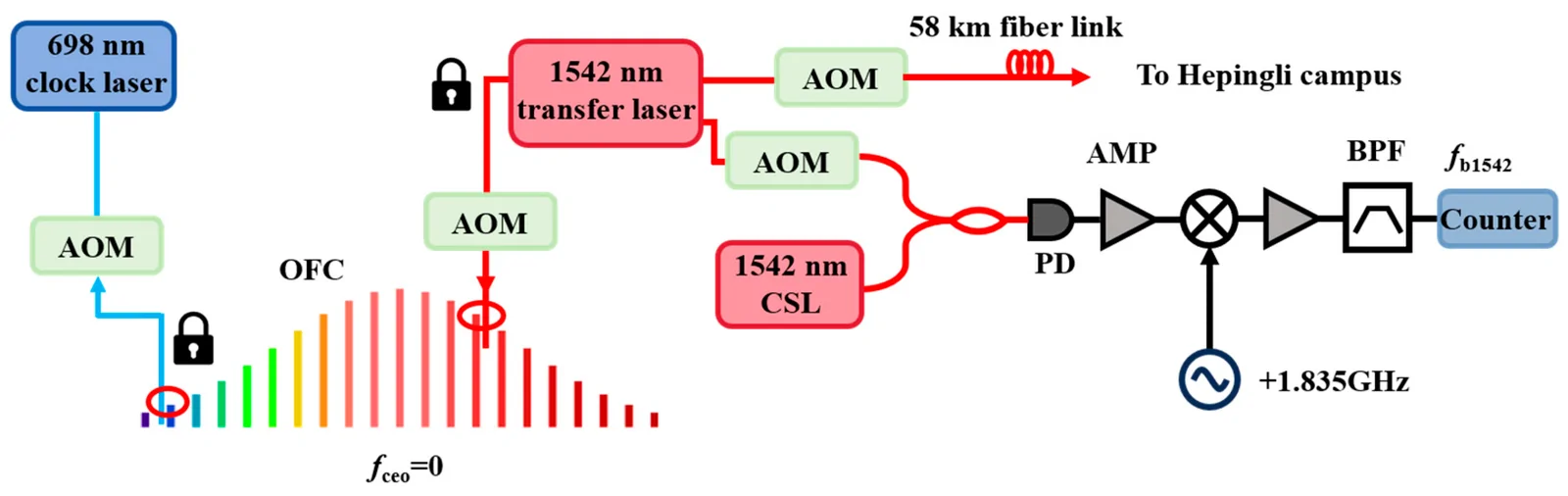
Experimental setup for remote comparison of two optical clocks and determination of comb mode numbers at the Changping campus. OFC: optical frequency comb; AOM: acousto-optic modulator; CSL: cavity-stabilized laser; PD: photodetector; AMP: amplifier; BPF: band-pass filter.

The experimental setup at the Changping campus for remote comparison between two optical clocks is shown in Figure 1. The optical frequency comb (DFC CORE, TOPTICA Photonics SE, Munich, Germany) works as a clockwork to transfer the stability of the 698 nm clock laser to a transfer laser at 1542 nm for remote comparison [22]. The optical frequency comb utilizes a difference frequency generation technique to passively stabilize its *f*_ceo_ with a comb spacing of 200 MHz [25]. Owing to the negligible frequency uncertainty of *f*_ceo_, utilizing this type of OFC allows us to omit *f*_ceo_ terms in calculations [26,27]. The comb has a dual-wavelength common-mode output of 698 nm and 1542 nm. The 698 nm clock laser and the 1542 nm cavity-stabilized laser (Stable Laser Systems, Boulder, CO, USA) beat with the comb, generating beat notes with >30 dB signal-to-noise ratio (SNR) in a 100 kHz resolution bandwidth (RBW).

**Figure 2:**
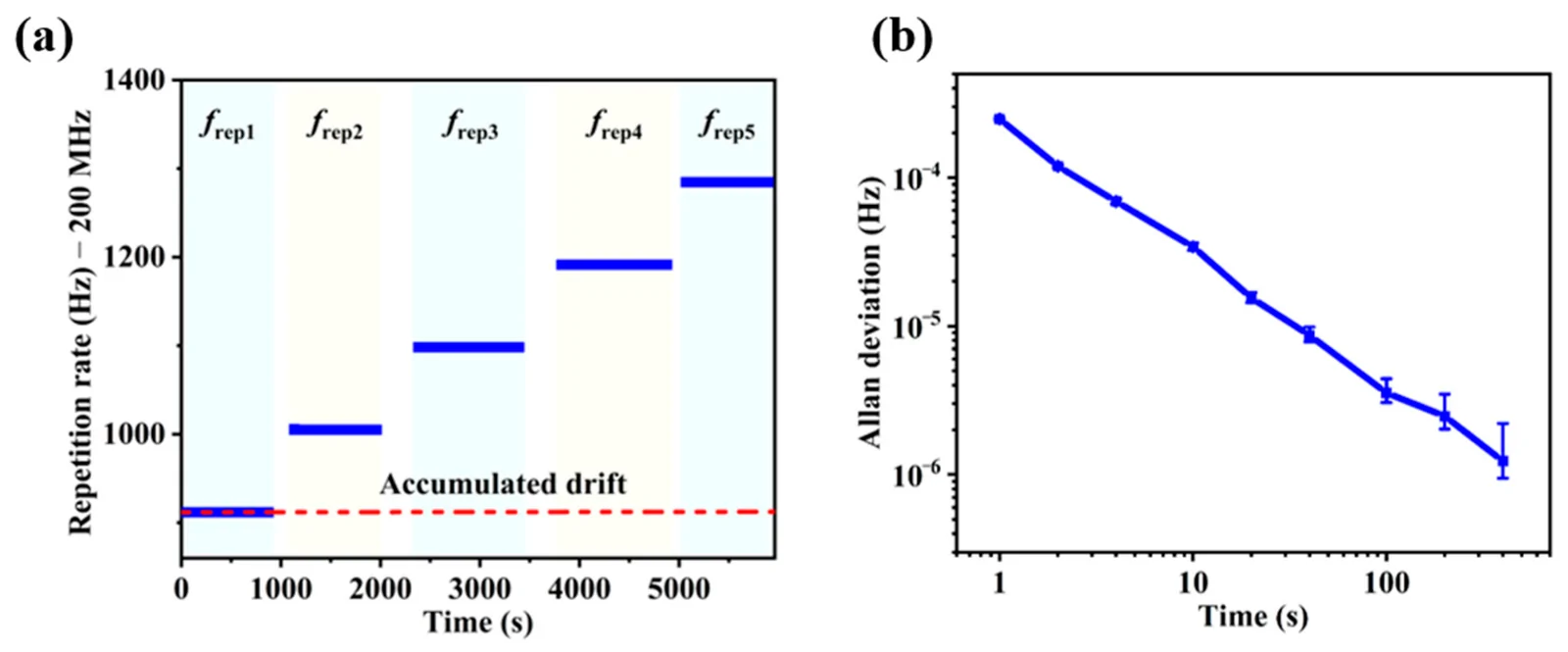
(**a**) Representative recorded repetition rate. Data points during repetition rate switching are omitted for clarity. (**b**) ADEV of the recorded repetition rate.

The OFC is first referenced to the 698 nm clock laser, while its repetition rate is recorded by a frequency counter (FXE 08, K + K Messtechnik GmbH, Braunschweig, Germany). Figure 2a provides the recorded repetition rate, which undergoes a step increase when the comb mode that is locked to the 698 nm clock laser is switched to a nearby one. The recorded repetition rate has a linear drift owing to the frequency drift of the 698 nm clock laser. To eliminate this, the accumulated linear drift in each dataset of repetition rate needs to be manually removed. Substituting the average value of the repetition rate from each dataset into Equations (6)–(9) in Section 2 yields the numbers of the comb modes that the 698 nm laser beats, which are 2,146,130, 2,146,129, 2,146,128, and 2,146,127. The resulting absolute frequency of the laser is 429,228,006,919,536 (6) Hz at the initial measurement time. Although each dataset of repetition rate is recorded for >1000 s with a 1 s gate time in our case, the typical Allan deviation (ADEV) of the repetition rate shown in Figure 2b indicates that averaging for >10 s is sufficient to determine the correct comb mode number according to Equation (10).

**Figure 3:**
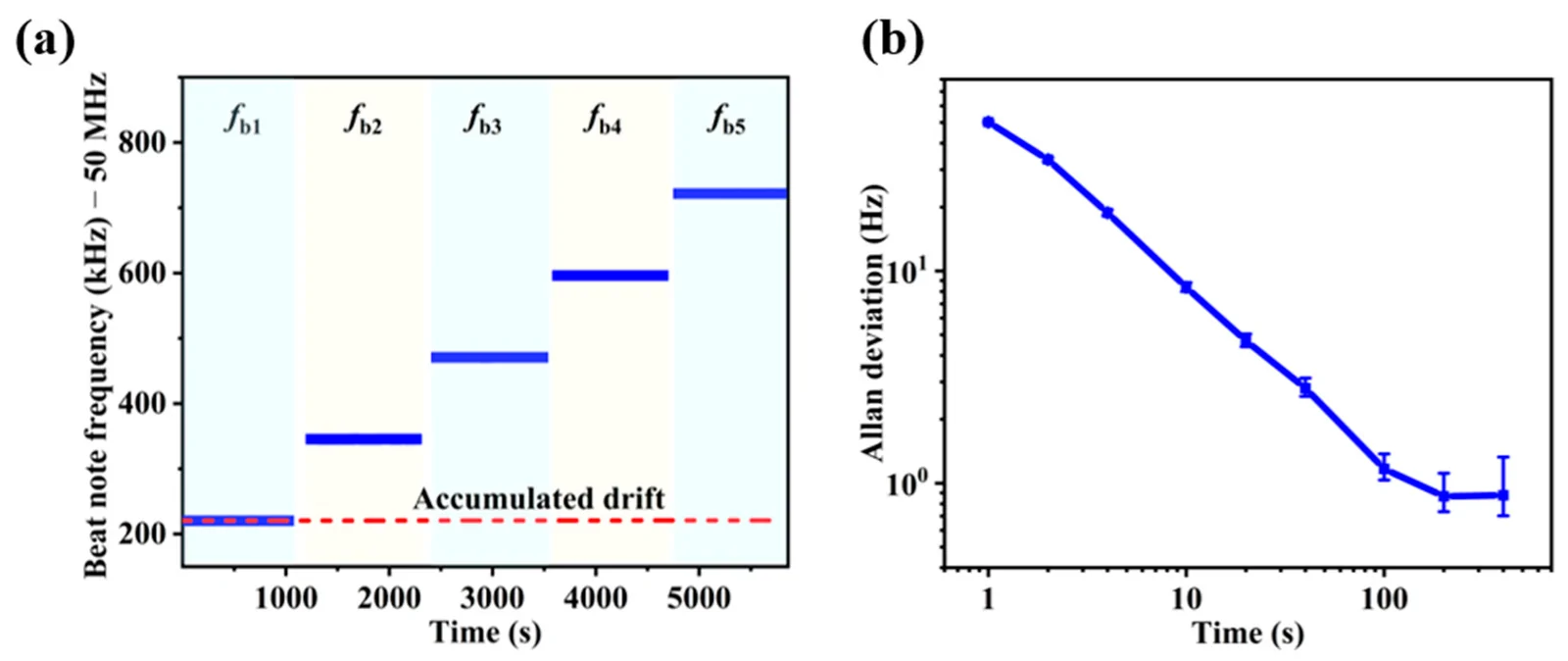
(**a**) Representative recorded comb-LUT beat. Data points during repetition rate switching are omitted for clarity. (**b**) ADEV of the recorded comb-LUT beat.

The repetition rate of the OFC is then locked to a frequency synthesizer that is referenced to a H-maser. The comb-LUT beat, observed when the 698 nm clock laser beats with five adjacent comb modes, is shown in Figure 3. These measurements are initiated at approximately *N* = 2,146,246 with the decrease in the comb’s repetition rate. The accumulated linear drift is removed, and the numbers of the comb modes that the 698 nm laser beats with are 2,146,216, 2,146,127, 2,146,128 and 2,146,129 according to Equations (16)–(19). The resulting absolute frequency of the laser is 429,228,006,919,210 (6) Hz at the initial measurement time. The typical ADEV of the comb-LUT beat shown in Figure 3b indicates that averaging for more than several seconds is sufficient to determine the correct comb mode number according to Equation (20).

The absolute frequency of the 1542 nm cavity-stabilized laser and the number of comb modes with which it beats can be determined using the same approaches as above. The comb mode numbers are 971,838, 971,837, 971,836 and 971,835 from Equations (6)–(9) and 971,834, 971,835, 971,836 and 971,837 from Equations (16)–(19), respectively. The absolute frequencies of this laser are 194,367,531,602,304 (5) and 194,367,531,594,327 (6) Hz from these two methods, respectively. The discrepancy in the two measurements is attributed to the linear frequency drift of the laser, estimated to be < 2 kHz/day, which occurred during the five-day interval.

During the comparison of the two optical clocks, the OFC is phase-locked to the 698 nm clock laser at a comb mode number of *N* = 2,146,216. The transfer laser is phase-locked to the OFC and subsequently transferred to Hepingli campus through a 58 km noise-canceled fiber link. Based on the known absolute frequency of the 1542 nm cavity-stabilized laser, the absolute frequency of the transfer laser at Hepingli campus can be determined locally at Changping campus. The transfer laser beats with the 1542 nm cavity-stabilized laser locally at Changping campus, generating a beat note at around 1.9 GHz, which exceeds the upper frequency limit of the counter. To resolve this, the beat note is mixed down to 50 MHz and subsequently measured by a frequency counter, as shown in Figure 1.

(21) $$f_{\mathrm{trans}}=f_{b1542}+f_{\mathrm{fs}}+f_{\mathrm{cs}}+f_{\mathrm{AOM}}$$

Following the equation above, the frequency of the transfer laser at Hepingli campus, *f*_trans_, is calculated to be 194,369,364,959,780 (5) Hz. The mode number of the Hepingli OFC’s comb that beats with the transfer laser is 777,440. *f*_b1542_ denotes the recorded average value of the down-mixed beat note, while *f*_fs_ represents the frequency of the signal generated from the frequency synthesizer for mixing. The absolute frequency of the 1542 nm cavity-stabilized laser is denoted as *f*_cs_, while *f*_AOM_ is the frequency shift introduced by the AOMs in the fiber links for noise cancelation. It is worth noting that the statistical uncertainty of *f*_b1542_ is 0.52 Hz. The statistical and systematic uncertainties introduced by the AOM and the electronic devices—such as the mixer, synthesizer, and counter—are evaluated to be at the μHz level, making them negligible [26,28]. The frequency of the transfer laser at Changping campus is calculated to be 194,369,417,459,780 (5) Hz. The mode number of the Changping OFC’s comb that beats with the transfer laser is 971,841.

## 4. Conclusions

In this work, we determine the numbers of OFC’s modes that beat with the clock laser and the transfer laser during long-distance optical clock comparisons locally at Changping campus, without the requirement of the simultaneous operation of the atomic systems, two OFCs, and a noise-canceled fiber link. To realize this, we obtain the numbers of comb modes and the absolute frequency of both the 698 nm clock laser and the 1542 nm cavity-stabilized laser by varying the repetition rate of the OFC. The absolute frequency of the transfer laser at Hepingli and Changping campuses is then derived by beating it against the 1542 nm cavity-stabilized laser, yielding values of 194,369,364,959,780 (5) Hz and 194,369,417,459,780 (5) Hz. Finally, the numbers of comb modes of both OFCs at Changping and Hepingli that beat with the transfer laser are determined to be 971,841 and 777,440, respectively.

As the Consultative Committee for Time and Frequency (CCTF) formally proposed mandatory criteria for redefining the second, which include an overall frequency ratio measurement uncertainty of less than 5 × 10^−18^ between optical clocks [29], our work mitigates the complexity of the simultaneous operation of two optical clocks, OFCs, and fiber link noise cancelation during long-distance comparison, thereby establishing a reliable foundation for remote clock frequency-difference calculations. Our work directly advances the evolving field of quantum sensing based on frequency comparison networks, thereby laying a vital groundwork for advanced sensing of fundamental physical quantities across diverse scenarios.

## Data Availability

The original contributions presented in this study are included in the article. Further inquiries can be directed to the corresponding author.
